# Optimal tracking control of the coal mining face fluid supply system via adaptive dynamic programming

**DOI:** 10.1038/s41598-023-47346-8

**Published:** 2023-11-15

**Authors:** Xiaoteng Zeng, Dalong Wang

**Affiliations:** 1 School of Mechanical Electronic and Information Engineering, China University of Mining and Technology – Beijing, Beijing, 100083 China; 2 Beijing Tianma Intelligent Control Technology Co., Ltd., Beijing, 101399 China

**Keywords:** Engineering, Mathematics and computing

## Abstract

Emulsion pump station is widely used to provide power for full-mechanized coal mining face equipment. With the development of the mining technique of longwall face and the extensive use of large mining height and high working resistance of the hydraulic support, the fluid supply pressure is more unstable and the pressure impact is increasingly intensified. The intelligent control of the emulsion pump station is the key to the development of the stable fluid supply technology at the working face. This paper develops a new on-line adaptive learning technique to improve the response speed of pumping station based on the optimal control algorithm of adaptive dynamic programming (ADP). The state space model of the working face fluid supply system in the process of no-load column lifting of hydraulic support is first established using the flow relationship, and the effectiveness of the proposed control algorithm is then verified by simulation validation. Moreover, combined with a certain fluid supply system of a fully mechanized caving face in Shandong Energy Xinwen Group XinJulong Co., Ltd, a technical scheme of adaptive stable fluid supply is verified in engineering practice. The results indicate that the fluid supply system using the proposed control algorithm has a favorable tracking effect on the ideal trajectory, and the tracking error can converge to near zero. The output pressure of the fluid supply system using the stable fluid supply technology is more stable, and the practical effect is good, which meets the demand of stable fluid supply in the mining face.

## Introduction

The fluid supply system of full-mechanized coal mining face consists of emulsion pump station, accumulator station, fuel tank, fluid supply and return pipeline and its accessories, etc. As the power source, the emulsion pump station can supply fluid and power for the hydraulic support of the working face^1^. With the increase of coal mining depth, hydraulic supports with large mining height and high working resistance are widely used, and the length of working face and the number of supports are increasing, which leads to the instability of fluid supply pressure and the increasingly aggravating pressure influence^2^.

It can only play an auxiliary role to improve the fluid supply circuit in the coal mining face and install an accumulator station in the fluid supply system in terms of unstable fluid supply pressure and intensified impact in the face. To fundamentally solve the above problems, it is necessary to adopt advanced algorithms to realize the intelligent control from the perspective of emulsion pump station, so as to supply fluid on demand and stabilize the system pressure. Therefore, according to the characteristics of the face, to improve the response speed of emulsion pump station, it is of crucial significance to design an adaptive control algorithm to make the hydraulic system more stable.

Intelligent control of emulsion pump station is the key to the development of the stable fluid supply technology in the coal mining face. In this respect, many control results are shown to emulsify pump such that Li et al.^3^ studied the fluid supply system at the working face and developed a distributed monitoring system to monitor the emulsion pump station in real time. Li and Wei^4^ calculated the number of hydraulic supports in operation according to the pressure characteristics and time history, so as to change the flow of the station and lay a theoretical foundation for multi-pump linkage control. Ting and Hong^5^ studied the coal mining face fluid supply system, and obtained a conclusion that controlling the start and stop of the emulsion pump based on pressure sensor can effectively reduce energy consumption. Zhao et al.^6^ proposed an intelligent variable frequency speed control system to reduce the energy consumption of the station. Tan et al.^7^ introduced a pressure control method of emulsion pump station based on Elman neural network (NN), and verified the effectiveness of the method through simulation and comparison. Wang and Li^8^ took the fluid supply system of Taran Gaole Coal Mine as the research object, and suggested using the control mode of frequency converter combined with combination switch to realize constant pressure fluid supply in the coal mining face. Qi^9^ prejudged the power demand of the support by predicting the haulage speed of the shearer, and then used the hybrid particle swarm multi-objective optimization algorithm to realize the intelligent adjustment of the pressure flow of the station. Wei et al.^10^ brought forward that by establishing the connection between the full-mechanized mining fluid supply system and the electro-hydraulic control system of the hydraulic support, the fluid demand for the face can be predicted and the fluid can be supplied on demand. By making simulation analysis of the intelligent constant pressure fluid supply system, Mei Sun pointed out that the system has a vital influence on the performance of the hydraulic support^11^. Fu et al.^12^ proposed a stabile-pressure fluid supply principle with reasonable flow in advance based on the operating characteristics of the support. Tian et al.^13^ studied the application of PLC in the frequency conversion and constant pressure control system of mine emulsion pump station, and founded that the system based on PLC can effectively reduce energy consumption. Li et al.^14^ studied the intelligent fluid supply technology in the coal mining face, and elaborated the key technologies such as intelligent on-demand fluid supply control technology and remote fluid supply technology.

To ensure the support effect of the hydraulic support and make the equipment movement at the working face more stable and accurate. Shu et al.^15^ drew a conclusion that the three-inlet and three-return fluid supply pipeline is beneficial to reduce the pressure loss. By constructing mathematical models of pipeline and load, Li et al.^16^ concluded that parameters such as pipe length and pipe diameter can affect the stability of hydraulic system. Zhao et al.^17^ used Matlab/Simulink to analyze the transient state of high-pressure pipeline and simulated the dynamic characteristics of pressure transient. Zhao^18^ built the simulation model of trapezoidal and three-inlet and three-return fluid supply pipeline, and concluded that the latter can reduce the pressure loss and improve the column lifting speed. Li^19^ analyzed the layout of emulsion pump station at the working face with large mining height and the circular fluid supply program, and verified its rationality through theoretical arithmetic. Wiens^20^ initiated a distributed gradient line tower model and conducted modeling analysis of the fluid pipelines, which ensure faster calculation speed and higher precision.

In order to further improve the stability of fluid supply in the coal mining face, Mamcic and Bogdevicius^21^ built a simulation model of hydraulic accumulator, and analyzed the influence of accumulator volume on its effect. Li et al.^22^ analyzed the influence of accumulator parameters on its performance, and verified it using experiments. Van de Ven^23^ created a new variable-area piston constant-pressure accumulator, which can keep the accumulator at a constant pressure during the oil output. Ding et al.^24^ studied the effect of accumulator absorbing pressure pulsation of hydraulic pump, and proposed methods to increase the available capacity of accumulator. Bi et al.^25^ studied the absorption law of accumulator to system pressure and flow fluctuation by combining the method of simulation and experiment. Wan et al.^26^ studied the influence of accumulator parameters and accumulator station configuration scheme on the stability of fluid supply system in the coal mining face, and formulated configuration optimization scheme for the specific working face.

It can only play an auxiliary role to upgrade the long-distance fluid supply circuit and configure an accumulator station in terms of improving the stability of fluid supply in the coal mining face. But it can reduce pressure loss, alleviate hydraulic shock, and compensate system flow to a certain extent. Using advanced intelligent control method to realize the real-time response of emulsion pump under different working conditions is the key to improve the stability of fluid supply in the face. However, the current intelligent fluid supply technology in the coal mining face cannot ensure the speed and stability. The researches on intelligent fluid supply technology are mostly limited to detecting the system pressure and adjusting the fluid supply of pumping station based on unloading valve control technology (multi-pump linkage), frequency conversion constant pressure fluid supply technology or their combination. As the multi-pump linkage has low control accuracy and frequency conversion and constant pressure has slow response, the fluid supply system cannot accurately provide the required power, and there remains a certain impact of fluid supply pressure and flow fluctuation, which affects the control accuracy of the coal mining face support system. Therefore, in light of the specific coal mining face and based on the optimal control algorithm via adaptive dynamic programming (ADP), this paper develops a new online adaptive learning technology to track the system state in real time, and adaptively adjust and output the corresponding stable fluid supply flow. By establishing the state space model of the fluid supply system, the proposed algorithm is simulated and validated, and the technical scheme of stable fluid supply is proposed using the actual working conditions of the working face, which provides reference for engineering practice.

## Optimal control via adaptive dynamic programming

For the optimal control of nonlinear systems^27,28^, it is not easy to solve Hamilton–Jacobi–Bellman (HJB) equation, thus, Werbos proposed ADP to solve the above problems^29^. The ADP structure based on single-layer critic NN can reduce computational complexity and realize optimal tracking control^30^.

In this paper, the tracking error and reference trajectory are used to construct the augmented system, and the modified HJB equation can be obtained by constructing the performance index function. To solve the HJB equation on-line, a new adaptive learning algorithm is proposed to directly learn the unknown critic NN weights. The calculation efficiency can be improved since only the critic NN is used.

### Problem description

Considering a continuous-time nonlinear system:

1 $$ \dot{x} = A(x) + B(x)u $$

where $x \in {\mathbb{R}}^{n}$ is the system states and *u* is the control input.

Assuming that the system in Eq. (1) is controllable and the tracked ideal trajectory $x_{d} \in {\mathbb{R}}^{n}$ is continuously, the purpose of this paper can be summarized as designing a controller *u* to make the system states *x* track the ideal trajectory *x*_*d*_ that is, $e_{t} = x - x_{d} \to 0$.

To realize the optimal tracking control, an augmented system is constructed based on the tracking error *e*_*t*_ and the ideal tracking trajectory *x*_*d*_. According to the system and the ideal tracking trajectory, we have

2 $$ \dot{e}_{t} = \dot{x} - \dot{x}_{d} = A(x) - \dot{x}_{d} + B(x)u $$

Define $X = \left[ {e_{t}^{T} ,x_{t}^{T} } \right]^{T} \in {\mathbb{R}}^{2n}$, the augmented system can be obtained as

3 $$ \dot{X} = \left[ \begin{gathered} A(x) - \dot{x}_{d} \\ \dot{x}_{d} \\ \end{gathered} \right] + \left[ \begin{gathered} B(x) \\ 0 \\ \end{gathered} \right]u\mathop = \limits^{\Delta } F(X) + G(X)u $$

with $$F(X) = \left[ \begin{gathered} A(x) - \dot{x}_{d} \\ \dot{x}_{d} \\ \end{gathered} \right]$$, $$G(X) = \left[ \begin{gathered} B(x) \\ 0 \\ \end{gathered} \right]$$.

Based on the above operations, we can consider the tracking control as the regulation control problem of the augmented system (3).

In order to realize the optimal control of the system, a control *u* should be found to minimize the following performance index function:

4 $$ J(X) = \int_{t}^{\infty } {\left[ {X^{T} (\tau )E_{T} X(\tau ) + u^{T} (\tau )Ru(\tau )} \right]} d\tau $$

where $E_{T} = diag\left\{ {E,\left. {0_{n \times n} } \right\}} \right.$, $E \in {\mathbb{R}}^{n \times n}$ and $R \in {\mathbb{R}}^{m \times m}$ are the weight matrices of augmented system states *X* and control input *u* respectively.

The derivative of the performance index function (4) along the augmented system state is

5 $$ \dot{J}(X) = X^{T} E_{T} X + u^{T} Ru $$

According to the optimal criterion, the Hamiltonian function can be derived as

6 $$ H(X,u,J_{X} ) = X^{T} E_{T} X + u^{T} Ru + J^{T}_{X} (F(X) + G(X)u) $$

where* JX* = *∂J/∂X*.

Define the optimal performance index function J*(X) as

7 $$ J^{*} (X) = \mathop {\min }\limits_{u} J(X) $$

then the corresponding HJB equation can be obtained as

8 $$ 0 = \mathop {\min }\limits_{u} H(X,u^{*} ,J^{*}_{X} ) $$

Set $\partial H(X,u^{*} ,J^{*} )/\partial u^{*} = 0$, then we can obtain the solution of the optimal control

9 $$ u^{*} (X) = \frac{1}{2}R^{ - 1} G^{T} (X)\;\;J^{*}_{X} (X) $$

### Adaptive optimal control design

Because of the J^*^_X_ in (9) is unknown, this section will estimate the unknown J^*^_X_ based on ADP. To this end, a new HJB equation by combining the critic NN is constructed. To obtain the solution of HJB equation, an adaptive law based on parameter estimation error is designed to update the NN weights.

The core of ADP is to estimate the optimal performance index function J^*^(X) by using single-layer critic NN. Assuming that the performance index function J^*^(X) is continuous, the optimal performance index function J^*^(X) is:

10 $$ J^{*} (X) = W^{T} \sigma (X) + \varepsilon_{v} (X) $$

where $W \in {\mathbb{R}}^{l}$ is the ideal NN weight, $\sigma (X) \in {\mathbb{R}}^{l}$ is the activation function, *l* represents the number of neurons, and $\varepsilon_{v} (X)$ represents the NN approximation error.

The derivative of $J^{*} (X)$ with respect to *X*

11 $$ J^{*}_{X} (X) = (\nabla \sigma (X))^{T} W + \nabla \varepsilon_{v} (X) $$

where $\nabla \sigma (X) = \partial \sigma (X)/\partial X \in {\mathbb{R}}^{l \times 2n}$ and $\nabla \varepsilon_{v} (X) = \partial \varepsilon_{v} (X)/\partial X \in {\mathbb{R}}^{2n}$ are the partial derivatives of activation function and NN approximation error to *X*, respectively.

We assume *W*, σ, and $\nabla \varepsilon_{v}$ are bounded, that is, $\left\| W \right\| \le W_{M}$, $\left\| \sigma \right\| \le \sigma_{N}$, $\left\| {\nabla \sigma } \right\| \le \sigma_{M}$, and $\left\| {\nabla \varepsilon_{v} } \right\| \le \delta_{\varepsilon }$, the *WM, σN, σM, δε* are all positive constants.

In fact, the ideal NN weight *W* is unknown, therefore, the approximate performance index function is:

12 $$ \hat{J}(X) = \hat{W}^{T} \sigma (X) $$

where $\hat{W}$ is the estimated NN weight, which can be considered as the actual NN weight. Its derivative is:

13 $$ \hat{J}_{X} (X) = (\nabla \sigma (X))^{T} \hat{W} $$

The ideal optimal control can be obtained from Eqs. (10) and (11):

14 $$ u^{*} (X) = - \frac{1}{2}R^{ - 1} G^{T} (X)\left[ {\nabla \sigma^{T} (X)W + \nabla \varepsilon_{v} (X)} \right] $$

The actual optimal control can be obtained from Eqs. (12) and (13):

15 $$ u(X) = - \frac{1}{2}R^{ - 1} G^{T} (X)(\nabla \sigma (X))^{T} \hat{W} $$

In order to obtain the optimal control solution *u*, it is necessary to update the unknown critic NN weights *W* and guarantee its convergence, enabling the system output *x* to track the ideal trajectory *x*_*d*_. However, the adaptive learning algorithm used in most ADP methods can only ensure that the critic NN weights are uniformly and ultimately bounded (UUB), instead of converging to the ideal weights. Therefore, this paper proposes a new adaptive learning method to update the critic NN weights while ensuring convergence.

Substituting Eq. (11) into Eq. (8), a modified HJB equation can be obtained as follows

16 $$ X^{T} E_{T} X + u^{T} Ru + W^{T} \left\{ {\nabla \sigma \left[ {F(X) + G(X)u} \right]} \right\} + \varepsilon_{HJB} = 0 $$

where $\varepsilon_{HJB}$ is the approximate error of HJB equation.

To design an adaptive law to update the critic NN weights, the known term in the (16) is defined as

17 $$ \left\{ \begin{gathered} \Xi = \nabla \sigma (X)\left[ {F(X) + G(X)u} \right] \hfill \\ \Theta = X^{T} E_{T} X + u^{T} Ru \hfill \\ \end{gathered} \right. $$

Substituting (17) into (16), we can rewrite the HJB Eq. (16) as

18 $$ \Theta = - W^{T} \Xi - \varepsilon_{HJB} $$

According to Eq. (18), only the NN weight *W* is unknown. Therefore, it is necessary to design a learning algorithm, which is driven by the parameter estimation error, to learn the unknown weight solution.

Define filter matrices $O_{1} \in {\mathbb{R}}^{L \times L}$ and $E_{1} \in {\mathbb{R}}^{l}$

19 $$ \left\{ \begin{gathered} \dot{O}_{1} = - \ell O_{1} + \Xi \Xi^{T} ,O_{1} (0) = 0 \hfill \\ \dot{E}_{1} = - \ell E_{1} + \Xi \Theta ,E_{1} (0) = 0 \hfill \\ \end{gathered} \right. $$

where $\ell > 0$ represents a design parameter. Then the solution of Eq. (19) can be obtained

20 $$ \left\{ \begin{gathered} O_{1} = \int_{0}^{t} {e^{ - \ell (t - \tau )} \Xi (\tau )\Xi^{T} (\tau )d\tau } \hfill \\ E_{1} = \int_{0}^{t} {e^{ - \ell (t - \tau )} \Xi (\tau )\Theta (\tau )d\tau } \hfill \\ \end{gathered} \right. $$

We construct the auxiliary variable *θ* as

21 $$ \theta = \hat{W}O_{1} + E_{1} $$

From Eqs. (18) and (20), we have that $E_{1} = - O_{1} W + v_{1}$, among which $v_{1} = - \int_{0}^{t} {e^{ - \ell (t - \tau )} \varepsilon_{HJB} (\tau )\Xi } (\tau )d\tau$ is a bounded variable, which means, $\left\| {\nu_{1} } \right\| \le \varepsilon_{{{\text{v1}}}}$, and $\varepsilon_{{{\text{v1}}}} > 0$.

It can be obtained from Eqs. (19)–(21) that

22 $$ \theta = - \tilde{W}O_{1} + \nu_{1} $$

where $\tilde{W} = W - \hat{W}$ is the estimation error of NN weights.

The use of estimation error in the adaptive learning algorithm can ensure the convergence of the weights of the estimated NN. Therefore, the following adaptive laws can be designed to calculate $\hat{W}$ on-line: the estimated

23 $$ \mathop {\hat{W}}\limits^{.} = - \Gamma \theta $$

where $\Gamma > 0$ is the adaptive learning gain.

When the regression vector satisfies the continuous excitation condition, the matrix *O*_*1*_ is positive definite. Therefore, a Lyapunov function $Z_{1} = \tilde{W}^{T} \Gamma \mathop {\tilde{W}}\limits^{.} /2$ is selected, and its derivative to the adaptive law (23) is

24 $$ \dot{Z}_{1} = - \tilde{W}^{T} \Gamma^{ - 1} \mathop {\tilde{W}}\limits^{.} = - \tilde{W}^{T} O_{1} \tilde{W} + \tilde{W}^{T} v_{1} $$

then

25 $$ \dot{Z}_{1} = - \tilde{W}^{T} O_{1} \tilde{W} + \tilde{W}^{T} v_{1} \le - \left\| {\tilde{W}} \right\|(\kappa \left\| {\tilde{W}} \right\| - \varepsilon_{v1} ) $$

Therefore, the estimation error ${\tilde{W}}$ will converge exponentially to a small range, that is, $\Omega :\left\{ {{\tilde{W}}} \right.\left| {\left\| {{\tilde{W}}} \right.} \right.\left| \le \right.\varepsilon_{v1} /\left. \kappa \right\}$, and the extent depends on the NN estimation error *εv* and the excitation level *κ*. Ideally, when *εHJB* = 0, the estimation error ${\tilde{W}}$ converges exponentially to zero.

The proposed control method can be implemented on-line without any off-line learning. Moreover, the designed adaptive law can ensure that the critic NN weights converge to the neighborhood of its ideal weights. Based on the estimated NN weight $\hat{W}$, the actual control (15) can converge to the optimal control (14), i.e., $\left\| {\mu - \left. {\mu^{*} } \right\|} \right. \to 0$.

#### Remark 1

The adaptive law (23) is driven by the variable $\theta$ calculated from (22) with the auxiliary variables $\Xi$ and $\Theta$. As defined in (17), they can be calculated using the system output X for the regressor $O_{1}$ and output vector $E_{1}$. As shown in (21), $\theta$ contains information of estimation error $\tilde{W}$. Hence, the use of $\theta$ in the adaptive law can ensure that the estimation $\hat{W}$ converges to *W*. Hence, it outperforms the gradient based adaptive law, which was tailored for ADP synthesis.

## Fluid supply system model

**Figure 1:**
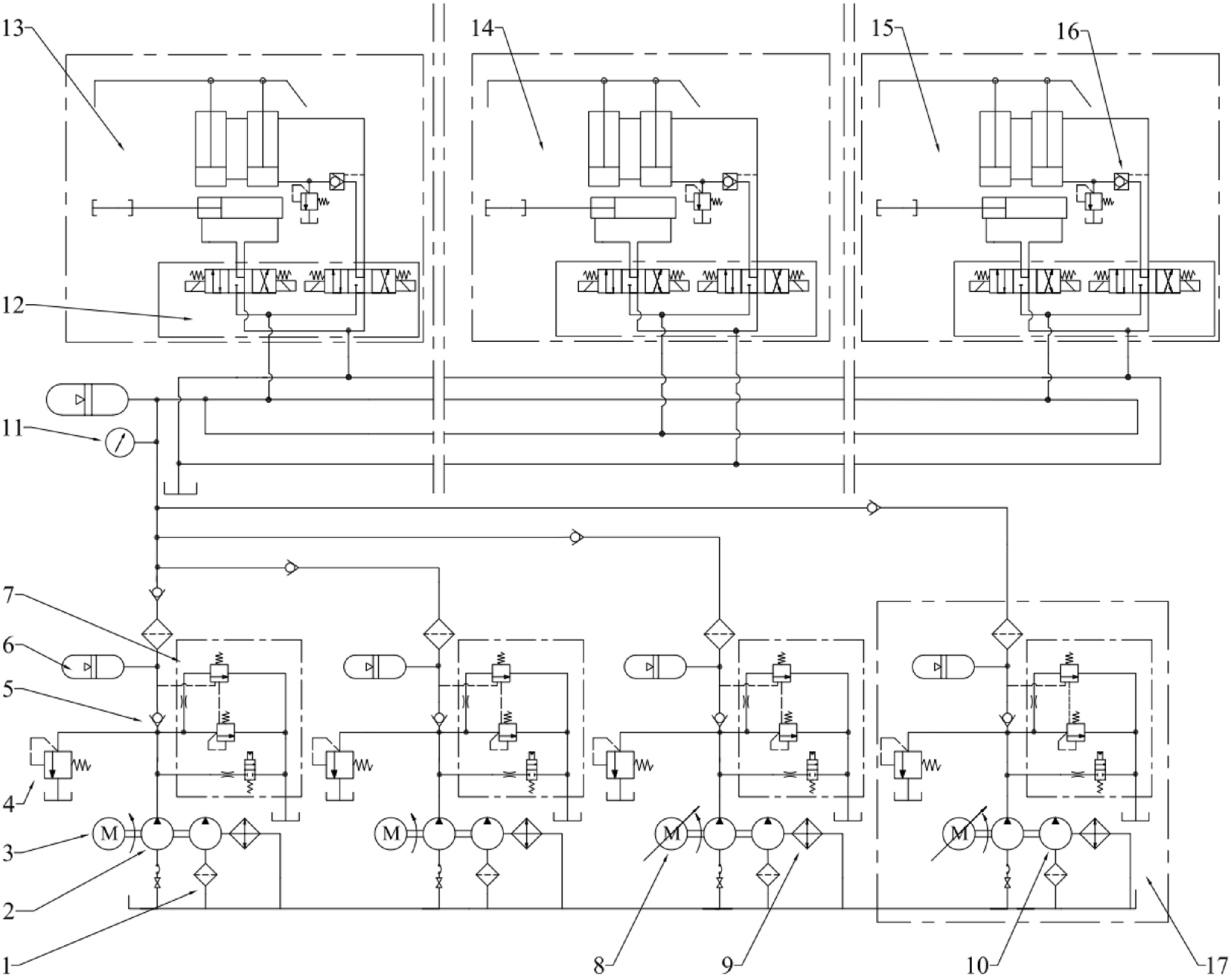
Schematic diagram of the emulsion pump station supply and configuration system for the coal mining face. 1- Filter; 2- Pump; 3- Motor; 4- Safety valve; 5-Check valve; 6- Accumulator; 7- Unloading valve; 8- Variable frequency motor; 9- Water cooler; 10- Gear pump; 11- Pressure gauge; 12- Reversing valve group; 13- First hydraulic support; 14-N hydraulic support; 15- Tail hydraulic support; 16- Pilot-operated check valve; 17- Backup variable frequency pump.

Emulsion pump station supplies fluid to hydraulic support and other equipment through fluid supply pipeline. Figure 1 shows the schematic diagram of the emulsion pump station supply system for the coal mining face. At present, frequency conversion constant pressure technology and multi-pump intelligent linkage technology based on unloading valve are widely used in pump station control. Taking a working face of XinJulong as an example, its emulsion pump station system adopts four emulsion pumps, two TMBRW(630/37.5)R power frequency emulsion pumps and two TMBRW(630/37.5)RA frequency conversion emulsion pumps, of which one is a main pump and the other is a standby.

In the process of safety support of the hydraulic support, the fluid demand of hydraulic support for lifting, pushing, lowering and pulling is different, and the way of support moving is quite different. Therefore, this paper studies with an example of a single support moving in sequence.

In order to match the speed of shearer, the support should finish a complete action flow every time the shearer passes through the width of a support. Then the relationship between the operating time of single hydraulic support and the speed of shearer is as follows:

26 $$ t_{z} = \frac{{60 \times L_{z} }}{{v_{c} }} $$

where *t*_*z*_ is the operating time of the single hydraulic support, which is s; *L*_z_ is the width of hydraulic support, which is m; *v*_c_ is the speed of shearer, which represents m/min.

The operating time of hydraulic support equals the sum of the time of lifting, pushing, lowering and moving, i.e.,

27 $$ t_{z} = t_{s} + t_{t} + t_{j} + t_{y} $$

In the operating process of hydraulic support, the fluid supply flow required for column lifting is the largest. However, the insufficient fluid supply often leads to deficient pressure, thus affecting the operating speed. Therefore, the fluid supply of a single hydraulic support is taken as the research object and other supports and related pipelines at the working face are ignored to analyze the column lifting process.

If the volume loss of emulsion pump station is ignored, the output flow is equal to the product of rotational speed and displacement, that is

28 $$ Q = mV_{m} $$

The output flow of emulsion pump can be divided into five parts:

29 $$ Q = Q_{0} + Q_{L} + Q_{c} + f + q $$

where *Q*_0_ represents the effective flow; *Q*_*L*_ represents the leakage in the column, the follow* Q*_*c*_ needs to be increased due to the compressibility of oil in hydraulic cylinder and pipeline; *f* is the system leakage, and *q* is the flow into (out of) the accumulator.

30 $$ Q_{0} = A_{1} \frac{dy}{{dt}} $$

where *A*_1_ is the piston area of the lower cavity of the column, and *y* is the piston displacement.

31 $$ Q_{L} = L\Delta P $$

where *L* is the leakage coefficient in the column, and *ΔP* is the pressure difference between the two sides of the piston.

32 $$ \Delta P = P_{1} - P_{2} $$

where *P*_1_ is the pressure of the lower cavity of the column, and *P*_2_ is the pressure of the upper cavity of the column.

When the column is undergoing no-load rising, *P*_2_, a constant, is equal to the back pressure of the fluid.

33 $$ Q_{c} = \frac{V}{4\beta }\frac{d\Delta P}{{dt}} $$

where *V* is the oil volume in the working cavity of the column and the oil inlet pipeline, and *β* is the effective volume elastic modulus of the system, which is 0.7–1.4 × 103 MPa.

In the fluid supply system of the working face, the total pressure loss is proportional to the *δ* power of the flow, that is

34 $$ \Delta P_{Z} = KQ^{\delta } $$

where *K* is the pressure loss coefficient, and *δ *= 2. According to the experimental data, there are some errors.

The pump station pressure *P*_2_ is:

35 $$ P_{0} = P_{1} + KQ^{2} $$

The system leakage is:

36 $$ f = L_{1} \cdot \frac{{P_{0} + P_{1} }}{2} = \frac{{L_{1} (2P_{1} + K(Q_{0} + Q_{L} + Q_{C} )^{2} )}}{2} $$

It is generally equipped with a large-capacity accumulator station in the emulsion pump station system, which is used to absorb pressure shock and pulsation, serve as an auxiliary power source, and so on. Its influence on the flow of the system is as follows:

37 $$ q = \frac{{V_{a0} }}{{Np_{a0} }}p_{a} $$

where *V*_*a*0_ serves as the initial volume of gas in the accumulator, $P_{a0}$is the initial pressure of gas in the accumulator, *N* is the gas polytropic index, and $P_{a}$ is the gas pressure in the accumulator.

Combining the above five equations, the following equations can be obtained:

38 $$ Q = A_{1} \frac{dy}{{dt}} + L\Delta P + \frac{V}{4\beta }\frac{d\Delta P}{{dt}} + f + q $$

$$mV_{m} = A_{1} \frac{dy}{{dt}} + L\Delta P + \frac{V}{4\beta }\frac{d\Delta P}{{dt}} + f + q$$

Simplifying:

39 $$ \frac{V}{4\beta }\frac{d\Delta P}{{dt}} + L\Delta P + f + q = mV_{m} - A_{1} \frac{dy}{{dt}} $$

The driving force of the piston is generated by the pressure difference between the two sides. The force balance equation is established by carrying out the force analysis of the piston:

40 $$ M\frac{{d^{2} y}}{{dt^{2} }} + c\frac{dy}{{dt}} = A_{1} \Delta P - f_{d} $$

Adding the auxiliary equation $c\dot{y} = c\dot{y}$:

42 $$ \left\{ \begin{gathered} \frac{V}{4\beta }\mathop {\Delta p}\limits^{.} = - A_{1} \dot{y} - L\Delta P - f - q + mV_{m} \\ c\dot{y} = c\dot{y} \\ M\dot{y} + c\dot{y} = A_{1} \Delta P - f_{d} \\ \end{gathered} \right. $$

Transforming to matrix form:

$$ \left[ \begin{gathered} \frac{V}{4\beta }\;\;\;0\;\;\;0 \hfill \\ \;\;0\;\;\;\;c\;\;\;0 \hfill \\ \;\;0\;\;\;\;c\;\;\;M \hfill \\ \end{gathered} \right]\left[ \begin{gathered} \mathop {\Delta P}\limits^{.} \\ {\dot{y}} \\ {\ddot{y}} \\ \end{gathered} \right] = \left[ \begin{gathered} - L\;\;\;0\;\;\; - A_{1} \hfill \\ \;\;0\;\;\;\;0\;\;\;\;\;c \hfill \\ \;A_{1} \;\;\;0\;\;\;\;\;0 \hfill \\ \end{gathered} \right]\left[ \begin{gathered} \Delta P \\ y \\ {\dot{y}} \\ \end{gathered} \right] + \left[ \begin{gathered} V_{m} \;\;\;0\;\;\;\;0 \hfill \\ \;0\;\;\;\;0\;\;\;\;0 \hfill \\ \;0\;\;\;\;0\;\;\; - 1 \hfill \\ \end{gathered} \right]\left[ \begin{gathered} m \\ 0 \\ f_{d} \\ \end{gathered} \right] + \left[ \begin{gathered} - f - q \\ 0 \\ 0 \\ \end{gathered} \right] $$

$$ \left[ \begin{gathered} \mathop {\Delta P}\limits^{.} \\ {\dot{y}} \\ {\ddot{y}} \\ \end{gathered} \right] = \left[ \begin{gathered} - \frac{4\beta L}{V}\;\;\;0\;\;\; - \frac{{4\beta A_{1} }}{V} \hfill \\ \;\;\;\;0\;\;\;\;\;\;0\;\;\;\;\;1 \hfill \\ \;\;\frac{{A_{1} }}{M}\;\;\;\;\;0\;\;\;\; - \frac{c}{M} \hfill \\ \end{gathered} \right]\left[ \begin{gathered} \Delta P \\ y \\ {\dot{y}} \\ \end{gathered} \right] + \left[ \begin{gathered} \frac{{4\beta V_{m} }}{V}\;\;\;0 \hfill \\ \;\;\;0\;\;\;\;\;\;0 \hfill \\ \;\;\;0\;\;\;\; - \frac{1}{M} \hfill \\ \end{gathered} \right]\left[ \begin{gathered} m \\ f_{d} \\ \end{gathered} \right] + \left[ \begin{gathered} - \frac{4\beta (f + q)}{V} \\ 0 \\ 0 \\ \end{gathered} \right] $$

Suppose the state vector $X = \left[ {\Delta P\;\;\;y\;\;\;\dot{y}} \right]^{T}$, the above formula can be rewritten as a state equation

43 $$ \dot{X} = AX + B\mu + D $$

where $$\dot{X} = \left[ \begin{gathered} \mathop {\Delta P}\limits^{.} \hfill \\ {\dot{y}} \hfill \\ \ddot{y}\; \hfill \\ \end{gathered} \right]$$, $$X = \left[ \begin{gathered} \Delta P \hfill \\ y \hfill \\ \dot{y}\; \hfill \\ \end{gathered} \right]$$, $$A = \left[ \begin{gathered} - \frac{4\beta L}{V}\;\;\;\;0\;\;\;\; - \frac{{4\beta A_{1} }}{V} \hfill \\ \;\;\;0\;\;\;\;\;\;\;\;0\;\;\;\;\;\;\;1 \hfill \\ \;\;\frac{{A_{1} }}{M}\;\;\;\;\;\;0\;\;\;\;\;\; - \frac{c}{M} \hfill \\ \end{gathered} \right]$$, $$B = \left[ \begin{gathered} \frac{{4\beta V_{m} }}{V}\;\;\;\;0\; \hfill \\ \;\;\;0\;\;\;\;\;\;\;0\; \hfill \\ \;\;\;0\;\;\;\;\; - \frac{1}{M} \hfill \\ \end{gathered} \right]$$, $$u = \left[ \begin{gathered} m\; \hfill \\ f_{d} \hfill \\ \end{gathered} \right]$$

$$ D = \left[ \begin{gathered} - \frac{4\beta (f + q)}{V}\; \\ 0 \\ 0 \\ \end{gathered} \right] $$

The output equation of the system is

44 $$ y = C_{1} X $$

where $$C_{1} = \left[ {0\;\;\;1\;\;\;0} \right]$$.

If *ΔP* is the system output,

45 $$ \Delta P = C_{2} X $$

where $$C_{2} = \left[ {1\;\;\;0\;\;\;0} \right]$$.

The above are the state space models of the fluid supply system.

## Simulation validation

According to the actual configuration of the system, the frequency conversion control of the pump station is realized based on the state space model built in “Fluid supply system model” section and the optimal control algorithm of ADP presented in “Optimal control via adaptive dynamic programming” section. The actual parameters of the system are substituted into the model to verify the effectiveness of the proposed algorithm.

According to the action characteristics of hydraulic support in the process of no-load column lifting and the actual situation of fluid supply system, the relevant parameters are calculated as follows:

$$  A =  \left[ {\begin{array}{*{20}c}    { - \frac{{4\beta L}}{V}} & {\quad 0} & {\quad  - \frac{{4\beta A_{1} }}{V}}  \\    0 & {\quad 0} & {\quad 1}  \\    {\frac{{A_{1} }}{M}} & {\quad 0} & {\quad  - \frac{c}{M}}  \\   \end{array} } \right]  = \left[ \begin{array}{ccc} - 2.489 \times 10^{ - 4} &\quad 0&\quad  - 2.533 \times 10^{8} \hfill \\ 0&\quad 0&\quad 1 \hfill \\ 4.0715 \times 10^{ - 5}&\quad 0&\quad - 0.01 \hfill \\ \end{array} \right]  $$

$$ B = \left[ \begin{gathered} 5.91 \times 10^{5} \\ 0 \\ 0\; \\ \end{gathered} \right.\;\;\;\;\;\;\;\left. \begin{gathered} 0 \\ 0 \\ - 1 \times 10^{ - 4} \\ \end{gathered} \right] $$

So:

$$ \dot{X} = \left[ \begin{gathered} - 2.489 \times 10^{ - 4} \Delta P - 2.533 \times 10^{8} \dot{y} \\ y \\ 4.0715 \times 10^{ - 5} \Delta P - 0.01\dot{y} \\ \end{gathered} \right] + \left[ \begin{gathered} 5.91 \times 10^{5} \\ 0 \\ 0\; \\ \end{gathered} \right.\;\;\;\;\;\;\;\left. \begin{gathered} 0 \\ 0 \\ - 1 \times 10^{ - 4} \\ \end{gathered} \right]\mu $$

The initial state of the system is:

$$ X = \left[ \begin{gathered} \Delta P \\ y \\ {\dot{y}} \\ \end{gathered} \right] = \left[ \begin{gathered} 245610 \\ 0 \\ 0 \\ \end{gathered} \right] $$

The ideal trajectory during the action is:

$$ X_{d} = \left[ \begin{gathered} \Delta P_{d} \\ y_{d} \\ \dot{y}_{d} \\ \end{gathered} \right] = \left[ \begin{gathered} 245610 \\ 0.03868t \\ 0.03868 \\ \end{gathered} \right] $$

If the state vector of the augmented system is $$\left[ \begin{gathered} \Delta P - \Delta P_{d} \\ y - y_{d} \\ \dot{y} - \dot{y}_{d} \\ \end{gathered} \right]$$, the state of the augmented system is:

$$ X = \left[ \begin{gathered} - 2.489 \times 10^{ - 4} \Delta P - 2.533 \times 10^{8} \mathop y\limits^{.} - 245610 \\ y - 0.03868t \\ 4.0715 \times 10^{ - 5} \Delta P - 0.01\mathop y\limits^{.} - 0.03868 \\ 245610 \\ 0.03868t \\ 0.03868 \\ \end{gathered} \right] + \left[ \begin{gathered} 5.91 \times 10^{5} \\ 0 \\ 0 \\ 0 \\ \;0\; \\ \;0\; \\ \end{gathered} \right.\;\;\;\;\;\;\;\left. \begin{gathered} 0 \\ 0 \\ - 1 \times 10^{ - 4} \\ 0 \\ 0 \\ 0 \\ \end{gathered} \right]\mu $$

To realize the optimal tracking control of the system, it is necessary to use NN reconstruction to minimize the performance index function. Therefore, the regression vector of the NN is designed as:

$$ \sigma = \left[ {X_{1}^{2} ,X_{2}^{2} ,X_{3}^{2} ,X_{4}^{2} ,X_{5}^{2} ,X_{6}^{2} } \right]^{T} $$

In the simulation validation, the initial NN weight is *0*, and the learning gain $\Gamma = 251$.

**Figure 2:**
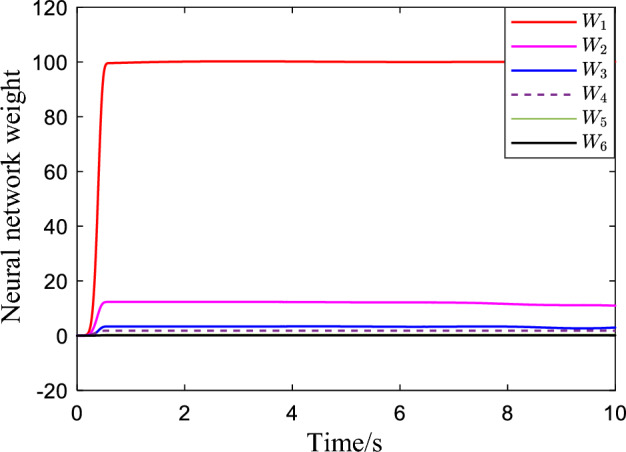
Profiles of critic NN weights.

**Figure 3:**
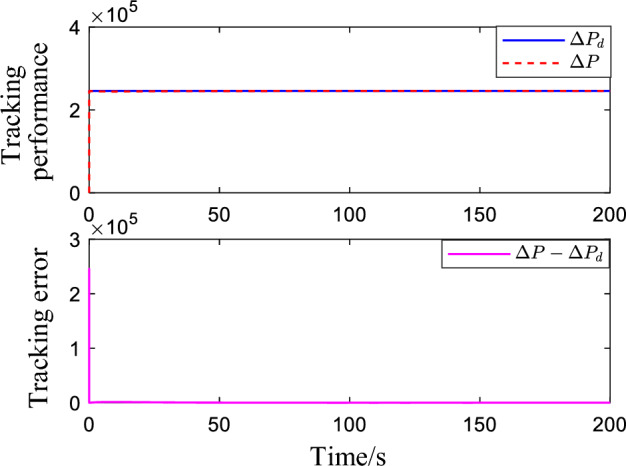
Pressure tracking performance.

**Figure 4:**
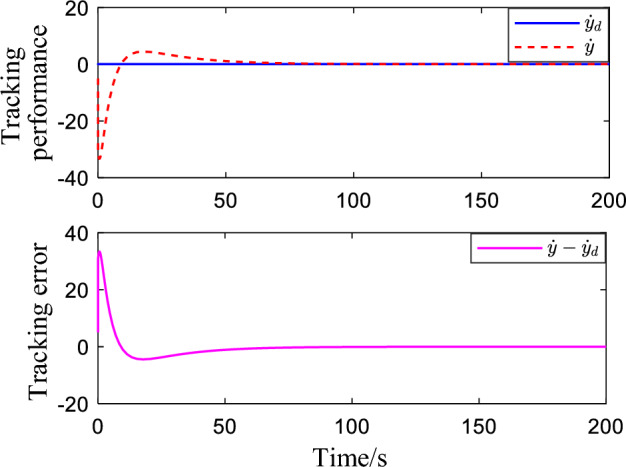
Speed tracking performance.

**Figure 5:**
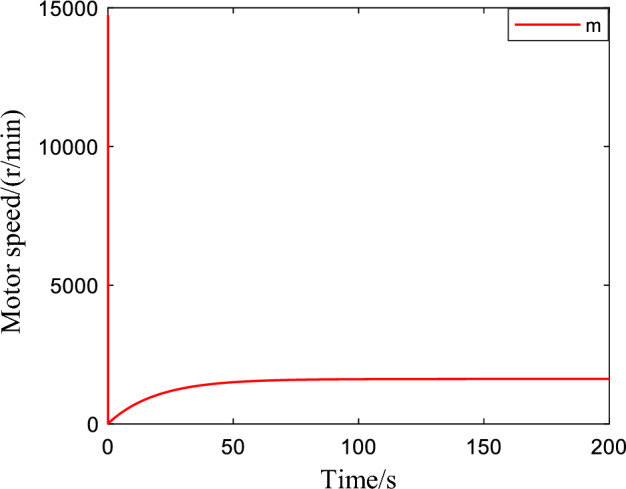
The speed of the pump.

Figure 2 shows the critic NN weights under the designed adaptive law learning. According to Fig. 2, the critic NN weights can converge to a specific value after a short time of on-line learning. Therefore, the weights obtained from on-line learning can be used for tracking control of the system. Figures 3 and 4 show the tracking performance and tracking error of the system on the ideal trajectory, respectively. The figures indicate that the system can adjust quickly and run according to the ideal trajectory, and the tracking error can be converged to near zero. Figure 5 shows the speed of the pump under the proposed optimal tracking control. In addition, it can be learned that the pump rotates stably and converges quickly with a favorable control effect.

**Figure 6:**
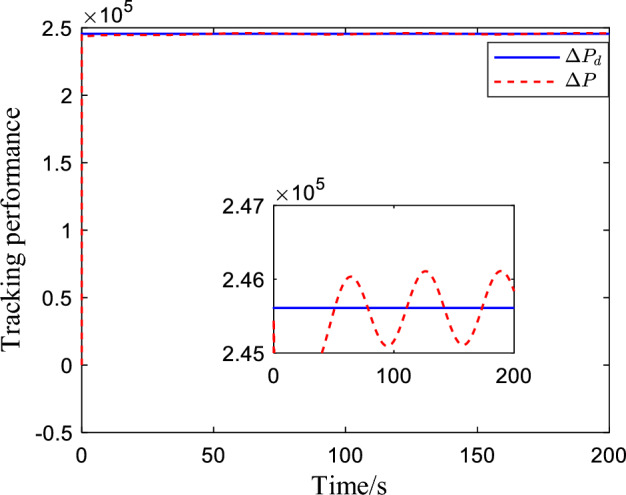
Pressure tracking performance when a disturbance is applied.

**Figure 7:**
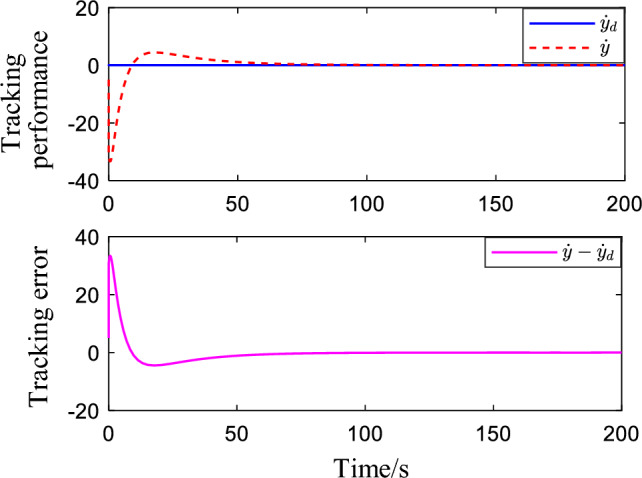
Speed tracking performance when a disturbance is applied.

In order to further verify the effectiveness of the proposed control algorithm, *ΔP* is disturbed to simulate the real working condition and make simulation analysis. Figures 6 and 7 show the tracking performance and tracking error of the system for the ideal trajectory respectively. It can be seen from the figures that the tracking error converges to near zero.

**Figure 8:**
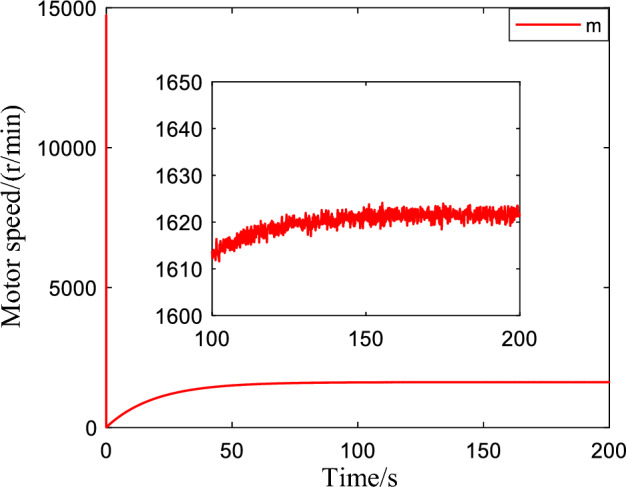
Pump speed when interference is applied.

**Figure 9:**
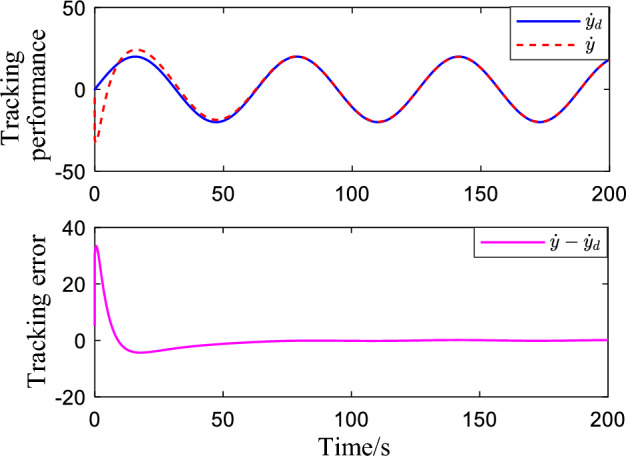
Speed tracking performance for complex signals.

Figure 8 illustrates the speed of the pump, where the speed of the pump tends to be stable and fluctuates around a certain constant value, which proves that the proposed control algorithm can adaptively adjust the speed according to the real state of the system. Figure 9 shows the tracking performance and tracking error of the system for sinusoidal signals. It indicates that the system has a good tracking performance, and the tracking error quickly converges to near zero. Therefore, the proposed control method is also suitable for complex signals.

These simulation results verify the effectiveness of the control method proposed in this paper.

## Engineering application of stable fluid supply technology at the working face

In view of the actual working conditions of a certain working face in XinJulong, considering the control mode of emulsion pump station, pipeline layout, accumulator configuration and other factors, the adaptive stable fluid supply technical scheme is proposed as follows:

1. To achieve the purpose of supplying fluid on demand and stabilizing the system pressure, the adaptive control method proposed in this paper is adopted to realize the intelligent adjustment of emulsion pump station, and accurately and timely match the fluid demand of hydraulic support.
2. To reduce the pressure loss and improve the response speed of the system, the working face adopts a network-type fluid supply system, the long-distance high-pressure hose for fluid supply is changed into a steel pipe, and the pipeline diameter is appropriately increased.
3. To further stabilize the system pressure, the fluid supply system at the working face uses an 8 × 63 L accumulator station, a small accumulator is added for each emulsion pump, and auxiliary accumulators are added at the end of the fluid inlet pipeline.

**Figure 10:**
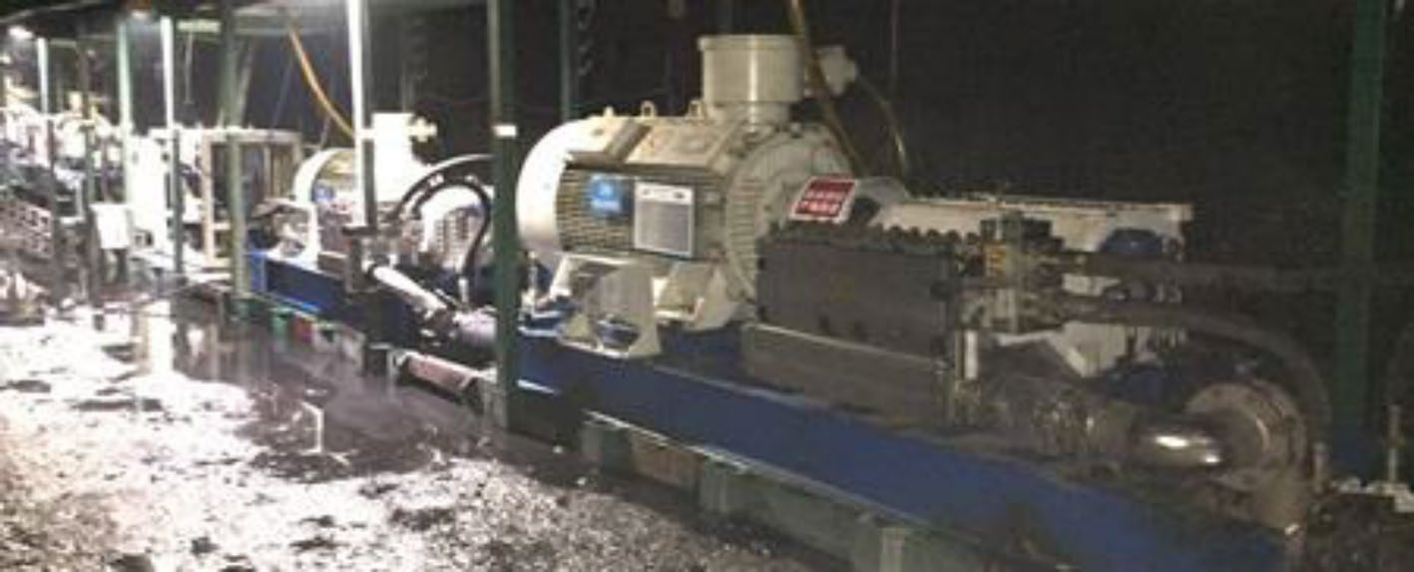
Application site of stable fluid supply technology for the coal mining face.

According to the above analysis, combined with the actual situation of the working face, the configuration of the working face is properly adjusted, and the output pressure of the emulsion pump station is monitored in real time by applying the adaptive stable fluid supply technology. The application site of the stable liquid supply technology in the working face is shown in Fig. 10.

**Figure 11:**
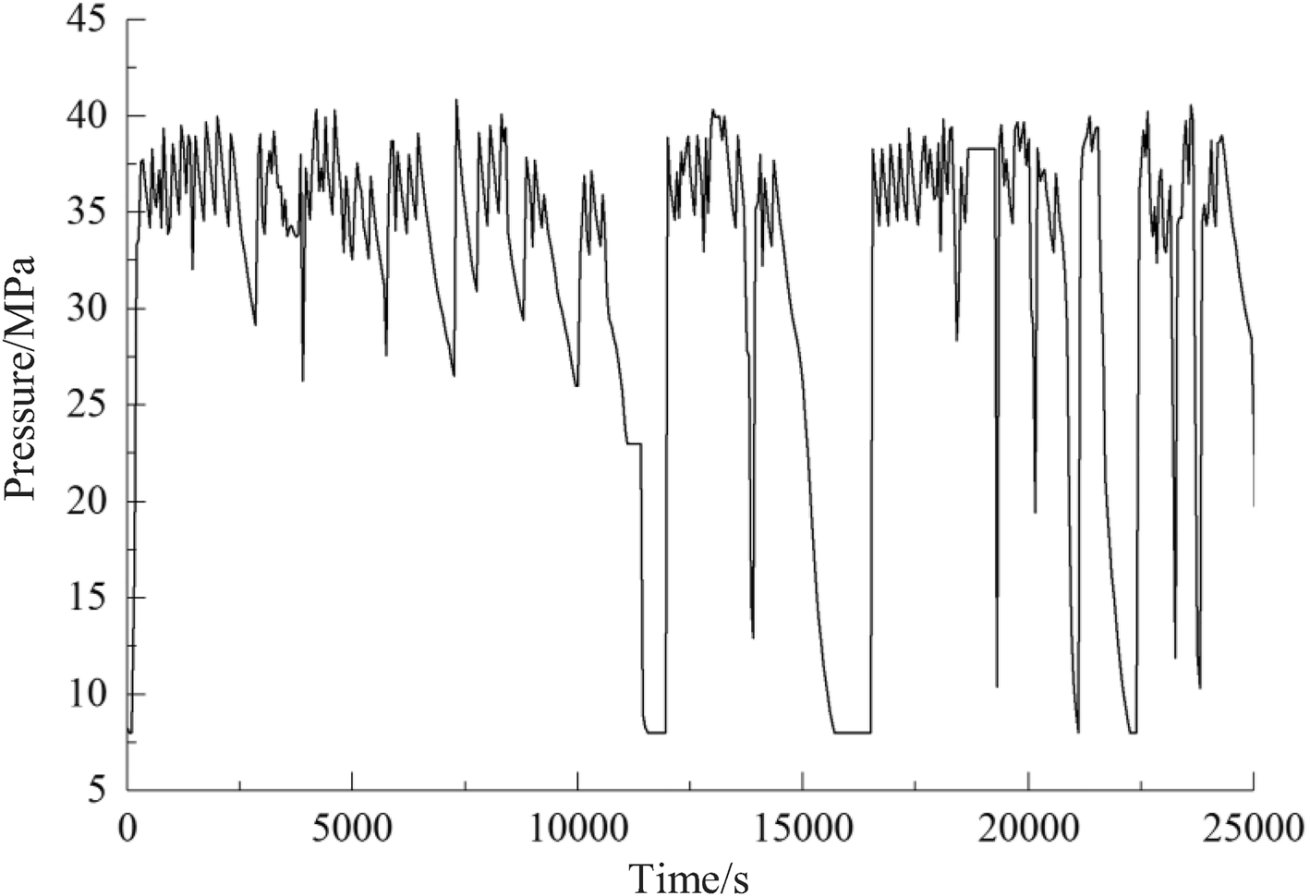
Comparison of pressure in pump station of original fluid supply system.

**Figure 12:**
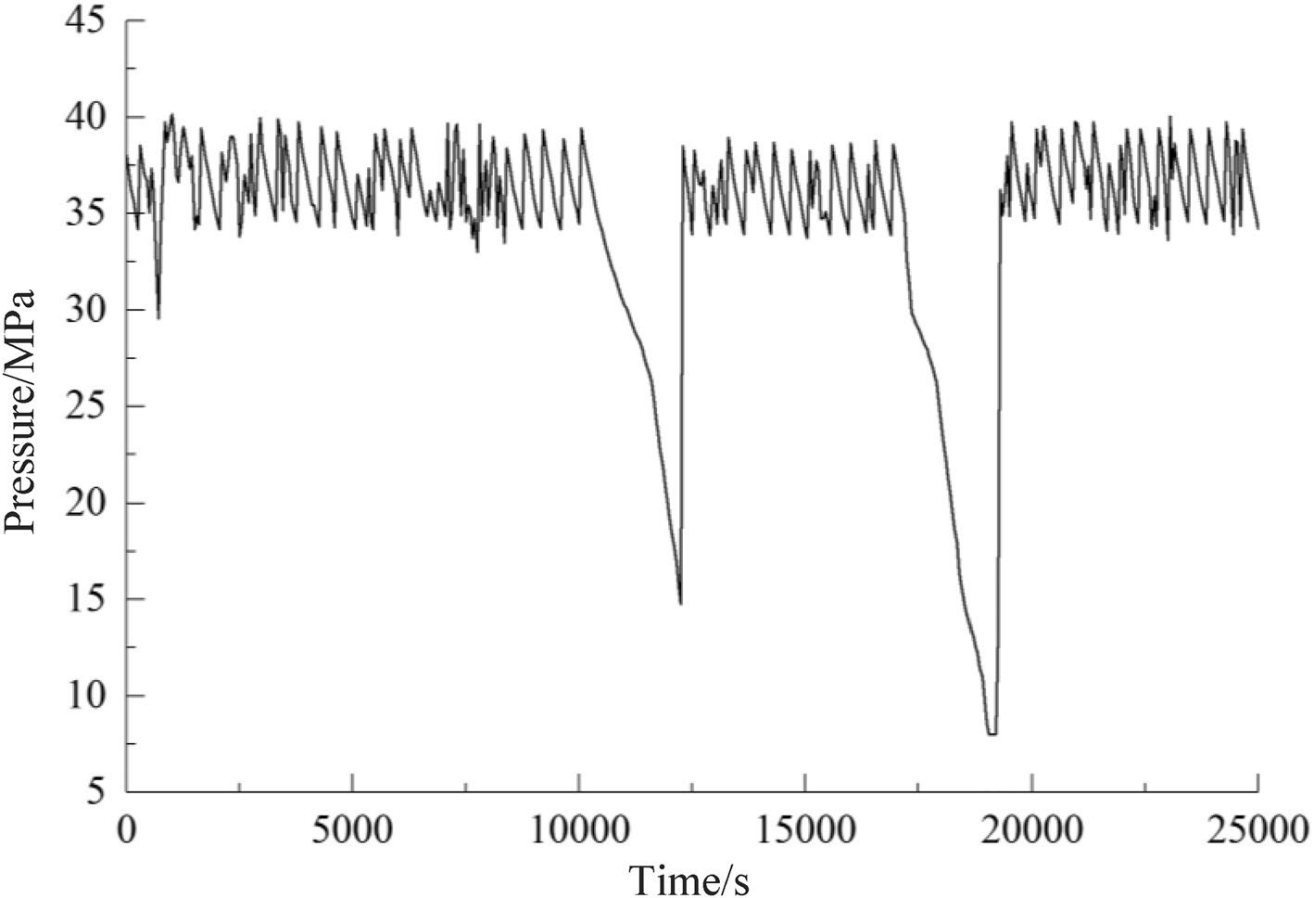
Comparison of pressure in pump station of stable fluid supply system.

According to Figs. 11 and 12, the system pressure fluctuates violently due to the different fluid consumption of the actuators such as hydraulic supports in the fluid supply system of the original working face. After the application of the adaptive stable fluid supply technology, the system pressure is relatively stable, fluctuating greatly only when the actuator uses a large amount of fluid, which shows that the adaptive stable fluid supply technology proposed in this paper can ensure the fluid supply on demand, improve the response speed, and stabilize the pressure of the system. The technology plays a crucial role in improving the performance of the hydraulic system at the working face and ensuring safe and efficient production in coal mines, and embraces promising practical application value.

## Conclusion

The control method of emulsion pump station in the fluid supply system of the working face is the key to realize the stable fluid supply technology of the working face. In this paper, a new on-line adaptive learning technique is proposed to design the optimal tracking control of the fluid supply system at the working face. In terms of specific working face, the technical schemes of the adaptive stable fluid supply are proposed and verified in engineering practice. The conclusions of this paper are as follows:

1. The optimal control algorithm based on ADP is described, and the augmented system is constructed based on the tracking error and ideal trajectory of the system output. In addition, the performance index function is constructed according to the tracking error and control input, and the critic NN is used for reconstruction to estimate on-line.
2. Through the flow relationship, the state space model of the fluid supply system in the working face is built by analyzing the action of hydraulic support lifting the column with no load driven by the fluid supply of the emulsion pump station, and comprehensively considering the factors such as the leakage in the column, the compressibility of emulsion, the leakage of pipeline system and the accumulator.
3. The proposed algorithm is applied to the fluid supply system for simulation validation, which shows that the system has a favorable tracking effect on the ideal trajectory, and the tracking error can converge to near zero.
4. According to the specific working face, the technical scheme of adaptive stable fluid supply is put forward and verified in engineering practice. By comparing the actual output pressure of the pump station, it can be concluded that the output pressure of the system is more stable after the application of the stable fluid supply technology, which meets the demand of stable fluid supply at the working face with a good practical performance.

## Data Availability

Data were curated by the authors and are available upon request. If necessary, you can contact the author Dalong Wang.
